# Expiratory rib cage compression in mechanically ventilated adults: systematic review with meta-analysis

**DOI:** 10.5935/0103-507X.20170014

**Published:** 2017

**Authors:** Lúcia Faria Borges, Mateus Sasso Saraiva, Marcos Ariel Sasso Saraiva, Fabrício Edler Macagnan, Adriana Kessler

**Affiliations:** 1 Programa de Residência Multiprofissional em Atenção em Terapia Intensiva, Universidade Federal de Ciências da Saúde de Porto Alegre e Irmandade Santa Casa de Misericórdia de Porto Alegre - Porto Alegre (RS), Brasil.; 2 Programa de Pós-Graduação em Ciências Pneumológicas, Universidade Federal do Rio Grande do Sul - Porto Alegre (RS), Brasil.; 3 Departamento de Fisioterapia, Universidade Federal de Ciências da Saúde de Porto Alegre - Porto Alegre (RS), Brasil.

**Keywords:** Physical therapy modalities, Respiratory therapy, Mucociliary clearance, Critical care

## Abstract

**Objective:**

To review the literature on the effects of expiratory rib cage compression on ventilatory mechanics, airway clearance, and oxygen and hemodynamic indices in mechanically ventilated adults.

**Methods:**

Systematic review with meta-analysis of randomized clinical trials in the databases MEDLINE (via PubMed), EMBASE, Cochrane CENTRAL, PEDro, and LILACS. Studies on adult patients hospitalized in intensive care units and under mechanical ventilation that analyzed the effects of expiratory rib cage compression with respect to a control group (without expiratory rib cage compression) and evaluated the outcomes static and dynamic compliance, sputum volume, systolic blood pressure, diastolic blood pressure, mean arterial pressure, heart rate, peripheral oxygen saturation, and ratio of arterial oxygen partial pressure to fraction of inspired oxygen were included. Experimental studies with animals and those with incomplete data were excluded.

**Results:**

The search strategy produced 5,816 studies, of which only three randomized crossover trials were included, totaling 93 patients. With respect to the outcome of heart rate, values were reduced in the expiratory rib cage compression group compared with the control group [-2.81 bpm (95% confidence interval [95%CI]: -4.73 to 0.89; I^2^: 0%)]. Regarding dynamic compliance, there was no significant difference between groups [-0.58mL/cmH_2_O (95%CI: -2.98 to 1.82; I^2^: 1%)]. Regarding the variables systolic blood pressure and diastolic blood pressure, significant differences were found after descriptive evaluation. However, there was no difference between groups regarding the variables secretion volume, static compliance, ratio of arterial oxygen partial pressure to fraction of inspired oxygen, and peripheral oxygen saturation.

**Conclusion:**

There is a lack of evidence to support the use of expiratory rib cage compression in routine care, given that the literature on this topic offers low methodological quality and is inconclusive.

## Abstract (pt)

**Objetivo:**

Revisar na literatura os efeitos da manobra de compressão torácica expiratória sobre a mecânica ventilatória, a desobstrução brônquica, e os índices de oxigenação e hemodinâmica de pacientes adultos ventilados mecanicamente.

**Métodos:**

Foi realizada uma revisão sistemática com metanálise de ensaios clínicos randomizados nas bases de dados MEDLINE (via PubMed), EMBASE, Cochrane CENTRAL, PEDro e LILACS. Foram incluídos estudos com pacientes adultos, internados em unidades de terapia intensiva, ventilados mecanicamente, que comparavam os efeitos da manobra de compressão torácica expiratória com grupo controle (sem manobra de compressão torácica expiratória) e que avaliaram os seguintes desfechos: complacência estática e dinâmica, volume de secreção depurado, pressão arterial sistólica, pressão arterial diastólica, pressão arterial média, frequência cardíaca, saturação periférica de oxigênio e relação entre pressão arterial de oxigênio e fração inspirada de oxigênio. Foram excluídos estudos experimentais com animais e estudos com dados incompletos.

**Resultados:**

A estratégia de busca resultou em 5.816 estudos, sendo incluídos apenas três estudos randomizados com *crossover*, totalizando 93 pacientes. No desfecho de frequência cardíaca, observou-se redução a favor da manobra de compressão torácica expiratória, comparada com o grupo controle [-2,81bpm (IC95%: -4,73 a 0,89; I^2^: 0%)]. Na complacência dinâmica, não foi observada diferença significativa entre os grupos [-0,58mL/cmH_2_O (IC95%: -2,98 a 1,82; I^2^: 1%)]. Nas variáveis, pressão arterial sistólica e pressão arterial diastólica após avaliação descritiva, foram encontradas diferenças significativas, entretanto, para variáveis volume de secreção, complacência estática, relação pressão arterial de oxigênio por fração inspirada de oxigênio e saturação periférica de oxigênio, não foram encontradas diferenças entre os grupos.

**Conclusão:**

Faltam evidências que sustentem o uso da manobra de compressão torácica expiratória na rotina assistencial, pois a literatura sobre o tema é de baixa qualidade metodológica e inconclusiva.

## INTRODUCTION

Critical patients hospitalized in intensive care units (ICUs) might need invasive ventilatory support for different reasons, including respiratory failure, acid-base imbalance, or to relieve ventilatory work.^(1)^ However, invasive mechanical ventilation also has deleterious effects caused by the endotracheal prosthesis, including changes in mucociliary clearance and inhibition of the coughing mechanism, which, in turn, favor areas of hypoventilation and atelectasis, thus increasing the risk of ventilator-associated pneumonia.^(2,3)^ Such complications lead to indications for physical therapy.^(4)^

Chest physical therapy consists of a set of interventions to improve respiratory mechanics and gas exchange by increasing the compliances of the respiratory system and the clearance of pulmonary secretion, thus easing proper pulmonary ventilation. Chest physical therapy is very important and is widely used among mechanically ventilated patients for both those who are intubated and those who are tracheostomized.^(3,5)^

Expiratory rib cage compression (ERCC), or squeezing,^(5)^ is among the most frequently used airway clearance techniques among adult critical patients.^(5,6)^ This technique consists of a manual thoracic compression applied during exhalation, followed by a release at the end of exhalation, aiming to increase expiratory flow, thus expanding the gas-liquid interaction and mobilizing secretions from peripheral to central regions, favoring their removal.^(5,6)^

However, scientific evidence remains scarce regarding the effects of ERCC on airway clearance in these patients. Some authors argue that ERCC does not lead to significant effects on the removal of secretions and respiratory mechanics.^(6-8)^

Due to its higher statistical power, a systematic review with meta-analysis of randomized clinical trials (RCTs) can provide more reliable estimates of the efficacy of treatment than clinical trials. Thus, the objective of the present study was to review the literature on the effects of ERCC on ventilatory mechanics, airway clearance, and oxygen and hemodynamic indices in mechanically ventilated adult patients.

## METHODS

### Eligibility criteria

RCTs on adult patients (aged 18 years and above) hospitalized in ICUs and mechanically ventilated were included. Studies comparing ERCC with a control group (without ERCC) and that evaluated pulmonary mechanics (dynamic and static compliance - Cdyn and Cst, respectively), oxygen indices (peripheral oxygen saturation - SpO_2_ and ratio of arterial oxygen partial pressure to fraction of inspired oxygen - PaO_2_/FiO_2_), airway clearance (sputum volume), and hemodynamic variables (systolic blood pressure - SBP, diastolic blood pressure - DBP, mean arterial pressure - MAP, and heart rate - HR) were selected.

Experimental studies on animals and those with incomplete data (which had no original full-text article and with no evaluation of the expected outcomes of this review) were excluded.

### Search strategy

A systematic review of RCTs was performed by searching for articles in the databases MEDLINE (via PubMed), EMBASE, Cochrane CENTRAL, Physiotherapy Evidence Database (PEDro), and *Literatura Latino-Americana e do Caribe em Ciências da Saúde* (LILACS; Latin American and Caribbean Literature on Health Sciences), in addition to a manual search in the references of published studies that matched the defined topic. Articles were narrowed down with the filters publication year (2000 to 2015), humans, adult, and no language restriction.

For each research platform, a specific strategy of crossing index terms or keywords was developed to retrieve topics in the scientific literature.

The search strategy used the following index terms: "respiratory therapy", "mucociliary clearance", "critical care", "artificial ventilation", and "breathing exercises", associated with a sensitive list of search terms for RCTs, which was developed by Robinson and Dickersin.^(9)^ The search strategy used for PubMed is shown in table 1.

**Table 1:** Search strategy used for PubMed

#5	(#4) AND (“2000”[Date - Create]: “2015”[Date - Create])
	
#4	(#1 AND #2 AND #3)
	
#3	(randomized controlled trial[pt] OR controlled clinical trial[pt] OR randomized controlled trials[mh] OR random allocation[mh] OR double-blind method[mh] OR single-blind method[mh] OR clinical trial[pt] OR clinical trials[mh] OR (“clinical trial”[tw]) OR ((singl*[tw] OR doubl*[tw] OR trebl*[tw] OR tripl*[tw]) AND (mask*[tw] OR blind*[tw])) OR (“latin square”[tw]) OR placebos[mh] OR placebo*[tw] OR random*[tw] OR research design[mh:noexp] OR follow-up studies[mh] OR prospective studies[mh] OR cross-over studies[mh] OR control*[tw] OR prospectiv*[tw] OR volunteer*[tw]) NOT (animal[mh] NOT human[mh])
	
#2	“Critical Care”[Mesh] OR “Care, Critical” OR “Intensive Care” OR “Care, Intensive” OR “Surgical Intensive Care” OR “Care, Surgical Intensive” OR “Intensive Care, Surgical”
	
#1	Respiratory Therapy”[Mesh] OR “Therapy, Respiratory” OR “Respiratory Therapies” OR “Therapies, Respiratory” OR “Physical Therapy Modalities” OR “Modalities, Physical Therapy” OR “Modality, Physical Therapy” OR “Physical Therapy Modality” OR “Physiotherapy (Techniques)” OR “Physiotherapies (Techniques)” OR “Physical Therapy Techniques” OR “Physical Therapy Technique” OR “Techniques, Physical Therapy” OR “manual therapy” OR “Chest compression” OR “compression therapy” OR “rib cage compression” OR “Mucociliary Clearance”[Mesh] OR “Clearance, Mucociliary” OR “Clearances, Mucociliary” OR “Mucociliary Clearances” OR “Mucociliary Transport” OR “Mucociliary Transports” OR “Transport, Mucociliary” OR “Transports, Mucociliary”

### Data analysis

This systematic review paper followed the recommendations proposed by the Preferred Reporting Items for Systematic Reviews and Meta-Analyses (PRISMA) Statement.^(10)^

The titles and abstracts of papers identified using the search strategy were evaluated by two researchers fully individually to guarantee personal independence during the process of paper selection. Paper abstracts that did not provide enough information regarding the inclusion and exclusion criteria were selected for full-text evaluation. In the second phase, the same reviewers evaluated the full papers separately and performed selections of the studies according to the eligibility criteria. Eventual disagreements between the researchers were solved by designating a third evaluator for a final recommendation.

### Assessment of risk of bias

The evaluation of methodological quality was performed in a descriptive manner, and the following characteristics of the included studies were considered: generation of a randomization sequence, concealed allocation, blinding of patients and therapists, blinding of outcome evaluators, intention-to-treat analysis, and description of losses and exclusions.^(11)^ This assessment was performed in an independent manner by the same two reviewers mentioned above.

Studies with no clear description of an adequate randomization and with no description of the concealing of allocation were considered as not informed. The use of intention-to-treat analysis was considered as confirmed in the evaluation for the studies in which the number of randomized participants and the number of analyzed participants were identical, except for those patients lost during follow-up or those who rescinded their consent to participate in the study. Studies without these criteria were considered not informed.

### Statistical analysis

The meta-analysis was performed by means of a random-effects model based on an inverse-variance approach.

Outcomes that could not be included in the meta-analysis have their results exhibited in a descriptive manner. Statistical significance was considered at alpha < 0.05. Statistical heterogeneity of treatment effects between studies was evaluated by means of Cochran's Q test and the inconsistency test (I^2^), and values above 25 % and 50 % were considered as indicative of moderate and high heterogeneity, respectively. All analyses were conducted using the software Review Manager 5.2 (Cochrane Collaboration).^(11)^

## RESULTS

**Figure 1:**
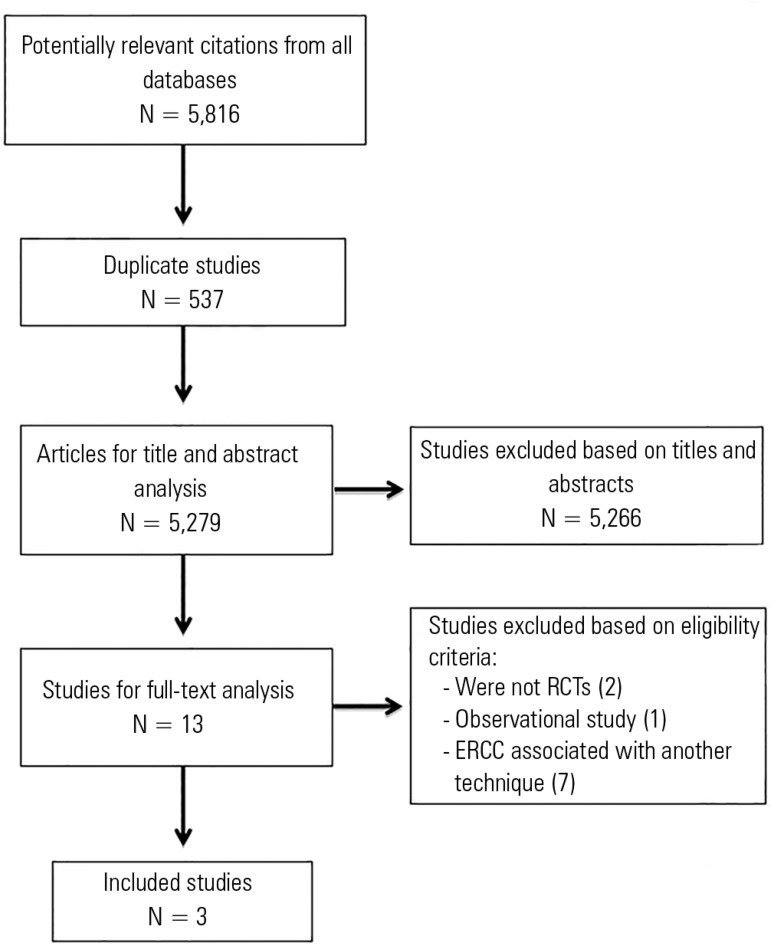
Flowchart of included studies. RCT - randomized clinical trial; ERCC - expiratory rib cage compression.

The search strategy produced 5,816 potentially relevant studies, of which 537 were excluded as duplicates, and 5,266 were excluded after reading the title and abstract. Only 13 studies met the eligibility criteria and were selected for full-text reading. Of these, three randomized crossover trials were included, totaling 93 patients. Two studies compared the strategy involving ERCC in addition to the usual care versus the usual care only (tracheal suctioning), and one study compared ERCC in addition to usual care versus positive end-expiratory pressure-zero end-expiratory pressure (PEEP-ZEEP) in addition to usual care. Figure 1 shows the flowchart of the studies included in this analysis, and table 2 summarizes the overall characteristics of the studies.

**Table 2:** Overall characteristics of the included studies

Study	Patients/age	Intervention	Outcomes
Unoki et al.^(6)^	31 subjects* 56,7 ± 17,60 years	IG: 5 min of ERCC performed by nurse before tracheal suctioning. Evaluation before intervention and at 25 min post-intervention CG: tracheal suctioning at 1 min and 25 min. Evaluation before intervention and at 25 min post-intervention	PaO_2_/FiO_2_ ratio Cdyn Secretion volume
Bousarri et al.^(12)^	50 subjects* 45,4 ± 18,14 years	IG: 5 min of ERCC before tracheal suctioning. Evaluation before intervention and at 15 min and 25 min post-intervention CG: suctioning at 1 min, 15 min and 25 min. Evaluation before intervention and at 15 min and 25 min post-intervention	SBP DBP HR
Santos et al.^(13)^	12 subjects* 54,9 ± 19,30 years	IG: 10 min of ERCC before tracheal suctioning. Evaluation before intervention and at 30 min post-intervention CG: PEEP-ZEEP before tracheal suctioning. Evaluation before intervention and at 30 min post-intervention	HR MAP Cdyn Cst SpO_2_

IG - intervention group; ERCC - expiratory rib cage compression; CG - control group; PaO_2_: partial pressure of arterial oxygen; FiO_2_: fraction of inspired oxygen; Cdyn - dynamic compliance of the respiratory system; SBP - systolic blood pressure; DBP - diastolic blood pressure; HR - heart rate; PEEP-ZEEP - positive end-expiratory pressure–zero end-expiratory pressure; MAP - mean arterial pressure; Cst - static compliance of the respiratory system; SpO_2_ - peripheral oxygen saturation.

* Intervention group = control group (data from the intervention group are the same as those from the control group).

### Risk of bias

Of the studies included in the systematic review, none exhibited the generation of an adequate randomization sequence and the blinding of patients, therapists, and outcome evaluators. Further, none of the studies used the principle of intention-to-treat for statistical analysis.

Two studies^(6,13)^ exhibited concealed allocation by using brown sealed envelopes and drawing for the definition of patient groups for interventions. Additionally, both of these studies^(6,13)^ described patient losses and exclusions in the course of follow-up (Table 3).

**Table 3:** Assessment of risk of bias

Studies	Unoki et al.^(6)^	Bousarri et al.^(12)^	Santos et al.^(13)^
Generated random sequence	No	No	No
Concealed allocation	Yes	No	Yes
Blinding of patients and therapists	No	No	No
Blinding of outcome evaluators	No	No	No
Description of losses and exclusions	Yes	No	Yes
Intention-to-treat analysis	No	No	No

### Effects of interventions

Among the selected studies, only that of Unoki et al.^(6)^ assessed the outcome volume of cleared secretion, which, after ERCC, was not higher than the volume obtained in the control group.

Further, Unoki et al.^(6)^ were the only ones to assess the PaO_2_/FiO_2_ ratio, showing that ERCC had no significant effect on this outcome compared with the control group. The study by Santos et al.^(13)^ evaluated SpO_2_, which also remained similar to that of the control group at 30 min post-ERCC. However, in the intragroup analysis, SpO_2_ was significantly increased in the ERCC group, rising from 96 % (94 - 98) to 98 % (95 - 100), p = 0.011.

**Figure 2:**
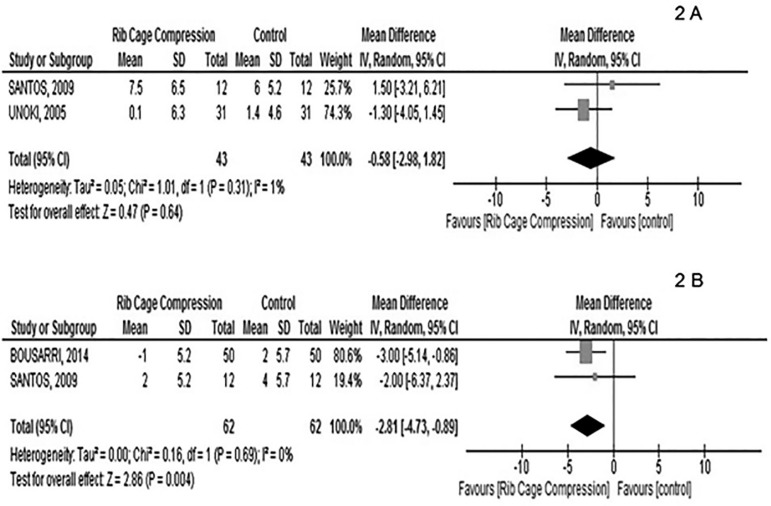
(A) Analysis of dynamic compliance after ERCC and PEEP-ZEEP^(13)^ and after ERCC followed by tracheal suctioning.^(6)^ (B) Analysis of heart rate after ERCC followed by tracheal suctioning^(12)^ and after ERCC and PEEP-ZEEP.^(13)^ ERCC - expiratory rib cage compression; PEEP-ZEEP - positive end-expiratory pressure-zero end-expiratory pressure.

Among the selected studies, two^(6,13)^ assessed Cdyn of the respiratory system, and both found that ERCC promoted no significant difference with respect to the control group. After the interpretation of the meta-analysis (Figure 2A), the difference between both studies on the effect of ERCC on the variable Cdyn was -0.58mL/cmH_2_O (95% confidence interval [95%CI]: -2.98 to 1.82). In these studies, the degree of inconsistency was low (I^2^: 1 %), indicating similarity between protocols.

The study by Santos et al.^(13)^ showed that Cst was increased at 30 min post-ERCC (pre versus post: 51.5mL/cmH_2_O (29 - 68) versus 62.0mL/cmH_2_O (36 - 71); p = 0.002), whereas the control group (PEEP-ZEEP) also exhibited a significant increase of Cst at 30 min after applying the technique (pre versus post: 49mL/cmH_2_O (34 - 69) versus 54.5mL/cmH_2_O (45 - 74); p = 0.002). However, in the analysis between groups, there was no significant difference in the effects of both techniques on Cst.

Two^(12,13)^ of the selected studies monitored HR (n = 62) before and after ERCC. In the post-maneuver measurement, HR was slightly but significantly reduced by 2.81 bpm compared with the control group (-2.81bpm; 95%CI: -4.73 to 0.89; I^2^: 0 %) (Figure 2B).

In the study of Bousarri et al.,^(12)^ SBP was significantly increased by 5mmHg and 3mmHg at 15 min and 25 min post-ERCC, respectively, whereas DBP was significantly increased by 3mmHg at 15 min post-ERCC. Interestingly, both SBP and DBP remained unchanged in the group submitted to tracheal suctioning only (control group). However, the effect of ERCC on hemodynamics seems transient, as values returned to baseline at 25 min post-intervention. On the other hand, in the study by Santos et al.,^(13)^ there were no significant changes in MAP after ERCC.

## DISCUSSION

### Evidence summary

Without a doubt, the main objective of ERCC is airway clearance. The most commonly used means of quantifying the efficiency of this technique is to measure the volume of cleared secretion. However, after the bibliographic survey for the present systematic review, it became clear that there is not enough evidence in the literature to support the use of this maneuver in mechanically ventilated patients, given that the volume of suctioned secretion was similar to that of the control group in all analyzed situations.

The build-up of secretion in the airways can negatively interfere with alveolar and capillary gas exchange. One commonly used means to gauge the exchange rate is to measure the PaO_2_/FiO_2_ ratio. In addition to this parameter, SpO_2_ also reflects the efficiency of gas exchange. Thus, another important aspect raised in this review is that these variables that provide information on the gas exchange rate remained similar in patients allocated to both groups. Further, no significant changes were found after ERCC with respect to parameters that are used to assess ventilatory mechanics (Cst and Cdyn).

Considering the high interaction between ventilatory and circulatory function, it is safe to assume that changes in the pulmonary system induce cardiovascular changes. To study this interaction, the hemodynamic variables must be assessed, which mainly include HR, SBP, DBP, and MAP. ERCC imposes external pressure on the rib cage. Nevertheless, the repercussion on the main hemodynamic variables was low. As shown in figure 2B, ERCC promoted a statistically significant reduction in HR. However, from a clinical standpoint, the magnitude of the change in HR was irrelevant, indicating that the maneuver imposes a low cardiovascular burden on patients under conditions similar to those studied. It is important to note that pressure changes also occur at a low-magnitude scale and in a transient manner, thus reinforcing the notion of safety attributed to ERCC.

However, in view of these findings, doubt remains regarding the benefits of ERCC for mechanically ventilated adult patients. At least for now, the choice of this physical therapy practice as a strategy to promote bronchial hygiene remains an unanswered question.

The study by Nozawa et al.^(14)^ characterized profiles of Brazilian physical therapists working in ICUs and revealed that physical therapy is predominantly characterized by applying techniques of pulmonary secretion removal and re-expansion (99.3%). These findings are in agreement with international studies, such as that of Berney et al.,^(15)^ which was conducted in Australia. Specifically, the study showed that 80% of the physical therapists used manual techniques for airway clearance.

As described previously, globally accepted techniques in clinical practice, such as ERCC, remain without reasonably sustained scientific evidence that justify their use, given that the attributed benefits are insufficient to eliminate doubts, leaving room for plenty of discussion in this matter.

Hence, this is the first systematic review with meta-analysis aiming to study the effects of ERCC on mechanically ventilated adult patients. As shown here, there is a lack of well-conducted studies objectively evaluating the effect of ERCC in the care of these patients; consequently, our results are inconsistent. Still, there is a high probability that ERCC, in the manner in which it is currently being applied, will provide few significant results in this population.^(6,12,13)^ This lack of significance is because each therapist has a different degree of strength and employs a varying time of execution (i.e., there is no established standardized minimum time for applying the technique).

### Comparison with other studies

Expiratory rib cage compression is a method for the removal of secretions that applies vigorous rib cage compression during exhalation, aiming to increase expiratory flow and to move secretions via mechanisms similar to those that occur during coughing. Using this technique, the physical therapist acts in easing secretion removal, which is then eventually concluded by means of coughing or tracheal suctioning.^(6,16)^ However, the effects of this maneuver on the removal of secretions and respiratory mechanics are controversial, possibly because the methodology does not follow a standardized protocol. This lack of confirmation can be easily verified with the literature that describes the concomitant use of ERCC and other techniques.^(6,8,17-19)^

Two studies have assessed the effects of ERCC on Cst and secretion volume in animal models.^(20,21)^ In one of these studies,^(21)^ the authors suggested that ERCC might produce an increase in Cst. However, this technique might promote atelectasis in this population due to the increased compression of the lung by the maneuver. This finding raises doubts with respect to the absence of hazards, suggesting the possibility of inducing adverse effects by the compression of lung structures that are vulnerable to collapse.

In the other study on an animal model, Martí et al.^(22)^ found that ERCC eased the removal of secretions compared with the control group. However, the study was performed on pigs that received a neuromuscular blocking agent, and the maneuver was performed for 15 min. Together, these two characteristics comprise an important issue to be considered, both with respect to the research protocols in animal research and in clinical practice on humans. Still, except in specific cases, the frequent use of neuromuscular blocking agents is not recommended in patients.

Importantly, in ERCC, there is an increase in expiratory flow during manual compression, and the eventual removal of secretions can trigger the cough reflex, often suppressed by the patient in an attempt to avoid the discomfort caused by the orotracheal tube.^(8)^

Another aspect that must be considered, in both experimental models and clinical practice, is the time of execution of ERCC, which is often described as less than 15 min.^(6,12,13)^

These two aspects, *i.e*., protective suppression of the cough reflex and the short time of ERCC application, might act together to limit the transport and clearance of pulmonary secretions. The low efficacy of this airway clearance technique would justify the lack of benefits in terms of the ventilatory mechanics of these patients. This lack of efficacy was shown in the results of the analysis of Cdyn, which exhibited no significant differences in any of the studied protocols.^(6,13)^ These findings are in agreement with a study by Guimarães et al.,^(8)^ which reported an increased expiratory flow after ERCC, though with no significant effects on secretion clearance. Additionally, the above study found no changes in Cdyn, thus corroborating the findings of the maneuver's low efficacy.

However, Gonçalves et al.^(16)^ concluded that ERCC promoted an improvement in Cst in the patient group that exhibited signs of bronchial obstruction due to secretions. In this study, the maneuver promoted airway clearance and an increase in Cst at 30 min post-ERCC. However, the authors found no improvements in the variables related to gas exchange.

Finally, ERCC, which is performed to accelerate the exit of air from the airways and, thus, to promote airway clearance, does not interfere significantly with hemodynamic variables, thus showing good tolerance and procedural safety from a cardiovascular standpoint. These factors are important considering that if the time of ERCC application is really a necessary condition to ensure effectiveness, then the cardiovascular system is possibly not a limiting factor, thus rendering an increase in the feasible time of ERCC application in future clinical trials. This possibility is very plausible, given that the present review found a low hemodynamic repercussion, observed essentially in the HR, which was reduced by 2.81bpm on average. However, the data on SBP, DBP and MAP were poorly consistent, though, in general, the repercussion was low and oscillated within a range from 3mmHg to 5mmHg, which fully reverted within a short recovery period after the interruption of the procedure. Normally, the hemodynamic changes observed after the lung secretion suctioning procedure are related to the stimulation of the vagus nerve, which subsequently reduces HR and MAP.^(23)^

These findings have already been shown previously by Yazdannik et al.,^(24)^ who found that HR was slightly increased when measured immediately after suctioning. However, this effect was transient, and at 3 min post-intervention, there was a slight and progressive reduction in HR, gradually returning to the values recorded under baseline conditions. Even if the information on the influence of ERCC on the hemodynamic state is scarce and poorly detailed, one can assume that the cardiovascular implications induced by ERCC are safe and well tolerated by stable mechanically ventilated adult patients from a cardiocirculatory standpoint.

### Strengths and limitations of the review

This study has several methodological strengths, such as formulating a specific research question, performing a sensitive, comprehensive and systematic bibliographic search, having explicit and reproducible eligibility criteria, having no language restriction and being performed by two reviewers independently, with adequate selection of the studies, data extraction, and analysis of methodological quality of the included papers, which was also performed by two reviewers, in addition to meta-analysis.

Heterogeneities were low (I^2^ = 1% and I^2^ = 0%), indicating similarity of the interventions, which increases the power of the study.

The RCTs included in this review were methodologically limited, given that none exhibited all of the items evaluated in the assessment of risk of bias. Further, the meta-analysis could not be performed for some outcomes since the analyzed studies exhibited diverging methodologies.

## CONCLUSION

This systematic review with meta-analysis suggests that treatment with expiratory rib cage compression promotes a reduction in heart rate, without changing dynamic compliance. However, the methodological quality of the included papers indicates that further randomized clinical trials are necessary in this field. Future studies must provide greater methodological accuracy and a larger patient sample. Thus, evidence is lacking to support the use of expiratory rib cage compression in mechanically ventilated adult patients since the currently available literature in this field is of low methodological quality and inconclusive.
